# CVAM: CNA Profile Inference of the Spatial Transcriptome Based on the VGAE and HMM

**DOI:** 10.3390/biom13050767

**Published:** 2023-04-28

**Authors:** Jian Ma, Jingjing Guo, Zhiwei Fan, Weiling Zhao, Xiaobo Zhou

**Affiliations:** 1 College of Electronic and Information Engineering, Tongji University, Shanghai 201804, China; 2 West China Biomedical Big Data Center, West China Hospital, Sichuan University, Chengdu 610041, China; 3 Med-X Center for Informatics, Sichuan University, Chengdu 610041, China; 4 West China School of Public Health and West China Fourth Hospital, Sichuan University, Chengdu 610040, China; 5 Center for Computational Systems Medicine, School of Biomedical Informatics, The University of Texas Health Science Center at Houston, Houston, TX 77030, USA; 6 McGovern Medical School, The University of Texas Health Science Center at Houston, Houston, TX 77030, USA; 7 School of Dentistry, The University of Texas Health Science Center at Houston, Houston, TX 77030, USA

**Keywords:** copy number alteration, HMM, variational graph convolutional autoencoder, spatial transcriptome

## Abstract

Tumors are often polyclonal due to copy number alteration (CNA) events. Through the CNA profile, we can understand the tumor heterogeneity and consistency. CNA information is usually obtained through DNA sequencing. However, many existing studies have shown a positive correlation between the gene expression and gene copy number identified from DNA sequencing. With the development of spatial transcriptome technologies, it is urgent to develop new tools to identify genomic variation from the spatial transcriptome. Therefore, in this study, we developed CVAM, a tool to infer the CNA profile from spatial transcriptome data. Compared with existing tools, CVAM integrates the spatial information with the spot’s gene expression information together and the spatial information is indirectly introduced into the CNA inference. By applying CVAM to simulated and real spatial transcriptome data, we found that CVAM performed better in identifying CNA events. In addition, we analyzed the potential co-occurrence and mutual exclusion between CNA events in tumor clusters, which is helpful to analyze the potential interaction between genes in mutation. Last but not least, Ripley’s K-function is also applied to CNA multi-distance spatial pattern analysis so that we can figure out the differences of different gene CNA events in spatial distribution, which is helpful for tumor analysis and implementing more effective treatment measures based on spatial characteristics of genes.

## 1. Introduction

Copy number alteration is one of the important characteristics of tumors. Theoretically, through the genome and transcriptome in the same cell, we can infer the molecular mechanism leading to the change in cell phenotypes and reconstruct the cell lineage tree to capture DNA mutation profiles obtained in the cell genome over time. Although many experts and scholars are committed to the research on the joint paired sequencing technology of DNA and RNA, it is still challenging to conduct high-throughput sequencing and unbiased analysis of DNA and RNA samples at the same time. Due to the technical barrier and sequencing cost, these sequencing technologies have not been widely developed. However, many existing studies have shown a positive correlation between the gene expression and gene copy number identified from DNA sequencing, providing a theoretical foundation for inferring the CNA of tumor cells from transcriptome data [1,2,3].

Nowadays, single-cell transcriptome sequencing has been widely used to analyze the heterogeneity in tumors [4,5]. Many tools based on transcriptome sequencing have also been developed to identify the changes in genomic copy numbers in cells. For example, inferCNV [6] identifies somatic large-scale chromosomal copy number alterations based on tumor scRNA-seq data through Bayesian and Hidden Markov Model (HMM) models. CaSpER [7] obtains profiles of cell genome copy numbers by combining gene expression and allele offset information. CopyKAT [8] uses the combination of GMM and chromosome breakpoint exploration with Markov Chain Monte Carlo to generate cell CNA profiles.

As we all know, traditional transcriptome sequencing will lead to the loss of cellular spatial information and destruction of the tumor microenvironment. In the molecular level analysis of genomics, tumor cells of the same clone exhibit local aggregation characteristics spatially, and the physical location of cells facilitates the analysis of tissue functions and corresponding pathological changes. In order to solve the spatial problem in gene sequencing, spatial transcriptome analysis technology has been developed in recent years [9]. We can not only analyze the spatial consistency and heterogeneity of tumor cells morphologically but can also explore the polyclonal characteristics of tumor cells and spatially related genes at the molecular level [10,11,12,13]. At present, there are still few tools for CNA inference for spatial transcriptome data. Although STARCH [14] integrates spatial information into CNA inference through HMRF, it can only infer the consistent CNA profile of each clone instead of each spot in spatial transcriptome data. Therefore, it is urgent to develop new tools to identify genomic variation as well as integrate genetic and transcriptional variation based on the spatial transcriptome.

In this study, we propose a machine learning tool, CVAM, to infer CNA profiles from spatial transcriptome data. This method consists of clustering analysis and CNA inference stages. For clustering analysis, the spatial information and gene expression information are fused to realize nonlinear dimensionality reduction by variational graph autoencoder (VGAE) with multi-tasks. Then, the unsupervised clustering of samples is implemented by the Louvain algorithm. In the CNA inference stage, the CNA profile of each spot is inferred by the HMM after multi-level filters based on the cell type information from the first stage. Compared with other methods, CVAM improves the performance of CNA inference. We also analyze the CNA event patterns through Ripley’s K-function based on the CNA profiles. In summary, CVAM applies current popular machine learning algorithms to the analysis and prediction of spatial transcriptome data, expanding the use of biological information from spatial transcriptome data. Moreover, it further promotes the widespread applications of relevant machine-learning algorithms in bioinformatics.

## 2. Materials and Methods

**Figure 1:**
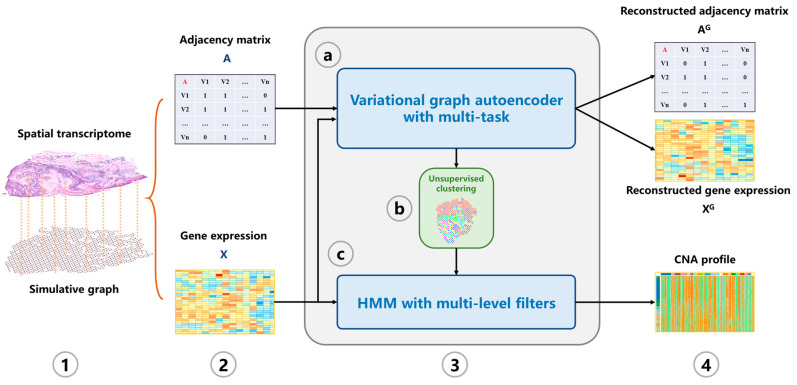
The overview of CVAM. (**1**) A graph is constructed according to the similarity and spatial information between spots. (**2**) The adjacency matrix and gene expression matrix of the graph are generated. (**3**) The CNA profile is inferred. (**3a**) The low-dimensional representation of spots in the hidden space is learned through the VGAE with multi-task. (**3b**) Spots are classified based on the learned representation. (**3c**) HMM with multi-level filters is used to infer the CNA profile. (**4**) The CNA profile of spots is output.

In this study, we propose a machine learning tool CVAM to infer CNA profiles of spatial transcriptomic data (Figure 1). First, a simulation graph corresponding to the spatial transcriptomic data is constructed. Then the hidden layer representations of spots are learned through multi-task VGAE. Subsequently, the spots are divided into different clusters based on the learned representation. According to the clusters, HMM is used to infer the genomic CNA profile of each spot.

**Figure 2:**
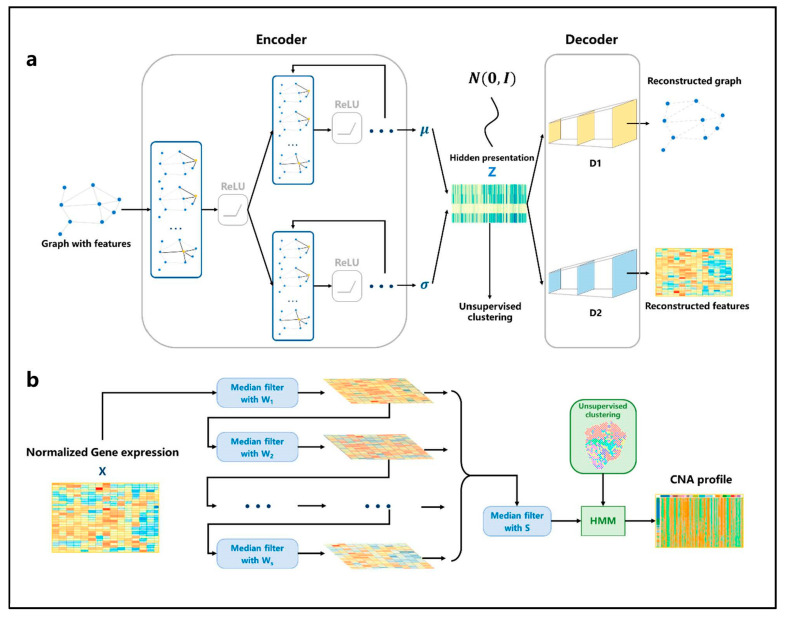
The architecture of VGAE and HMM. (**a**) The network structure of VGAE. For the input graph, the adjacency matrix and the gene expression matrix are aggregated through the single-layer GCN, and then the parameters of the distribution satisfied by the spots are learned through two GCN network modules. The hidden representation is generated through the reparameterization trick. Then, the adjacency matrix and the gene expression matrix are reconstructed through two fully connected neural networks. (**b**) Firstly, the gene expression profile is normalized, then multiple filters with different window sizes are used to smooth the gene expression data in order. Then, the smoothing results continue to be filtered through a median filter; finally, the results are input into the HMM model to infer the spots’ CNA profile according to the cluster.

The generation of the simulation graph is based on the spatial information of each spot and the similarity between spots (see Modeling). Based on the adjacency matrix and standardized gene expression matrix, the nonlinear low-dimensional representations of spots are learned through variational inference with an autoencoder (Figure 2a). First, for the input simulated graph, the adjacency information and gene expression information are aggregated through a shared GCN network layer. Then, two GCN neural network modules are constructed to learn the mean μ and variance σ of multivariate normal distribution satisfied by each spot in the hidden space, respectively. Then, through the reparameterization trick, the hidden space representation of each spot is obtained. Based on the hidden representation Z, two fully connected neural network modules are constructed to reconstruct the gene expression matrix and adjacency matrix of the graph. The Louvain algorithm is used to classify spots. Figure 2b shows the CNA inference process. The gene expression data are re-sorted according to the position of genes in chromosomes. After multi-media filtering, the spots are grouped based on the clustering results and HMM is applied to the data. Finally, the output is the genome CNA profile of each spot.

### 2.1. Modeling

#### 2.1.1. Construction of Graph

In order to introduce the spatial information of spots into the clustering, we construct the connection graph between spots according to the spatial coordinate information contained in the spatial transcriptome data and the similarity between spots. The graph is denoted by G. We use V to represent spots. First, we construct the graph through the k-nearest neighbor algorithm KNN and assign the weight $W_{1}$ to the edge with the connection between spots in the graph. Then, we calculate whether the connected spots are adjacent in spatial positions. If they are adjacent, $W_{2}$ is added to the weight of the edge. Finally, we construct the graph that integrates the spatial information and similar information of spots, and we use the adjacency matrix $A,\;A\in R{\;}^{n\times n}$ to represent the topology of each spot in the graph.

#### 2.1.2. The Architecture of VGAE with Multi-Task

Firstly, for the input reprocessed gene expression data $X^{\prime \prime }$ and the topological correlation adjacency matrix A, we use a k-layer graph convolution neural network(GCN) to fuse the spatial information and features of spots, namely,

$$GCN\left(X^{\prime \prime },\;Ã\right)=ÃReLU\left(ÃX^{\prime \prime }W_{0}\right)W_{1}$$

where $Ã=D^{-1/2\;}A\;D^{-1/2\;}$ is the symmetrically normalized adjacency matrix, and D is the degree matrix, recording the number of neighbor spots connected to each spot. Through $GCN_{\sigma }\left(X^{\prime \prime },\;Ã\right)$ and $GCN_{\mu }\left(X^{\prime \prime },Ã\right)$, the mean μ and variance σ of the normal distribution satisfied by each dimension of each spot in the hidden layer space were inferred. To simplify the parameter quantity of the model, the two modules share the first layer network parameters, and only the second layer network parameters are different. Moreover, to make each inferred dimension meet the standard normal distribution as much as possible, we compute the KL divergence between the inferred distribution and the standard normal distribution and add it to the loss function of the entire model for training and optimization. With the μ and σ, we can generate the corresponding low-dimension representation of each spot $Z\in R^{n\times d}$ through the reparameterization trick; here, d is the dimension of the hidden space. The formula can be represented as:

$$q\left(Z|X,A\right)=\sum_{i=1}^{n}q\left(z_{i}|X,A\right),\;with\;q\left(z_{i}|X,A\right)=N(z_{i}|\mu_{i},\;diag\left(\sigma_{i}^{2}\right))$$

For the hidden representation Z, we construct two fully connected neural network models respectively to accomplish two tasks (reconstruct the features of the spots and the spatial topology information):

$$D_{1}=W_{11}ReLU\left(W_{10}Z+b_{10}\right)+b_{11}$$

$$D_{2}=W_{21}ReLU\left(W_{20}Z+b_{20}\right)+b_{21}$$

On the one hand, it is to make the generated adjacency matrix $A^{G}$ as similar as the input adjacency matrix Ã if possible, that is, through $D_{1}$, the original spatial topology of the spatial transcriptome can be reconstructed as possible. In addition, we measure the difference between the generated matrix and the original matrix through BCE loss. On the other hand, with a network $D_{2}$, the gene expression matrix can be reconstructed, and we use MSE loss to measure the difference between the generated gene expression matrix $X^{G}$ and the input gene expression matrix $X^{\prime \prime }$. Compared with the decoder part of the traditional VGAE model, the reconstruction ability of our generated model is more robust.

#### 2.1.3. Loss Function

In order to balance the training loss of KL divergence and the reconstruction in the model, make the loss of them is made comparable, and avoid training the over-fitting model, we add a weight to each loss part to regularize the loss value, respectively. The loss function can be expressed as:

$$L=\lambda_{1}\cdot KL\left(q\left(Z|X,A\right)|N\left(0,I\right)\right)+\lambda_{2}\cdot BCE\left(Ã,A^{G}\right)+\lambda_{3}\cdot MSE\left(X^{\prime \prime },X^{G}\right)$$

namely,

$$L=\frac{\lambda_{1}}{2}\left(-\log\sigma^{2}+\mu^{2}+\sigma^{2}-1\right)+\frac{\lambda_{2}}{n}\sum_{i=1}^{n}\sum_{j=1}^{n}-{\tilde{A}}_{ij}\log A_{ij}^{G}+\frac{\lambda_{3}}{n\times m}\sum_{i=1}^{n}\sum_{j=1}^{m}{\left(X_{ij}^{\prime \prime }-X_{ij}^{G}\right)}^{2}$$

Finally, based on the Adam optimizer in Pytorch, the parameters are optimized by iteratively training the model through the back-propagation algorithm.

#### 2.1.4. Hidden Markov Model

After obtaining the hidden representation of each spot through the VGAE model, we use the Louvain algorithm to realize the unsupervised clustering of spots. After clustering, we use the Hidden Markov Model with multi-level filters to infer the CNA profile of each spot. First, we used a preprocessed gene expression matrix $\hat{X}$ according to the position of genes on the chromosome. The p-arm and q-arm of each chromosome is regarded as an independent sequence, and $S=\left\{x_{0},x_{1},x_{2},\ldots ,x_{i},\ldots ,x_{s}\right\}$ is used to represent the expression value of genes arranged along the p-arm or q-arm of a chromosome in the spot. For each sequence, we apply the median filter with window size W to smooth along the sequence. In order to reduce the dependence of W and improve the stability and effectiveness of the median filters, we use multi-level median filters to filter the initial gene expression sequence. Then, a median filter is used on these results after multi-level filters. Finally, HMM is used to infer the CNA profile by cluster based on clustering information. In this study, we mainly define three CNA states, including deletion, neutral, and amplification. Deletion means the gene copy number is 0 or 1. Neutral means diploid, while amplification means the gene copy number is more than two. We use $C=\left\{c_{0},c_{1},c_{2},\ldots ,c_{i},\ldots ,c_{s}\right\}$ to denote the gene CNA state along the sequence. $T={\left[t_{ij}\right]}_{3\times 3}$ is the transition probability matrix between the three states. The emission probabilities P of the three states meet the normal distribution with the mean value $\mu_{c}$ and variance $\sigma_{c}$, namely, $p(x_{i}|c_{i})=N\left(x_{i};\mu_{c},\sigma_{c}\right)$. In addition, π is the initial probability distribution of each state. The CNA states follow a first-order Markov chain and the CNA state of the current gene is only determined by the CNA state of the previous one, that is

$$P\left(c_{i}=a|c_{1},c_{2},\ldots ,c_{i}\ldots ,\;c_{s}\right)=P\left(c_{i}=a|c_{i-1}=b\right)\;with\;a,b\in \left\{\mathrm{Deletion},\;\mathrm{Neutral},\;\mathrm{Amplification}\right\}$$

Therefore, the probability model of S under parameters Φ in the HMM is expressed as:

$$P\left(S|\Phi \right)=\sum_{C}P(S|C,\Phi )P(C|\Phi ),\;with\;\Phi =\left\{T,\pi ,\mu_{c},\sigma_{c}\right\}$$

Next, the iterative solution of the model parameters can be realized by the Baum–Welch algorithm. Based on the estimated model parameters, the CNA profile C, which corresponds to the input gene expression value sequence S, can be inferred by the Viterbi algorithm.

### 2.2. Multi-Distance Spatial Pattern Analysis of CNA Event

In order to analyze the relationship between the CNA event and space, for the CNA profile of a gene in space, Ripley’s K-function is used to analyze the spatial point pattern of the CNA event from multi-distance. Ripley’s K-function is an index to describe the spatial structure of point processes under isotropic and mean conditions from multi-distance [15]. For a certain gene, each spot with CNA event of the gene is selected. As for these spots, the number of spots with the same CNA event in a circle with a radius d can be computed. Then, the results of all spots are summed up and divided by the total number of spots with CNA events as well as the density of the CNA event in the whole space. Namely:

$$K\left(d\right)=\frac{1}{\lambda }\sum_{i=1}^{N}\sum_{j=1,i\neq j}^{N}\frac{I_{d}\left(d_{ij}\right)}{N}\;,\;I_{d}\left(d_{ij}\right)=\left\{\begin{array}{c} 1,\;d_{ij}\leq d\\ 0,\;d_{ij}>d \end{array}\right.$$

Here, N is the number of spots with CNA events in the space and λ is the point density of the space.

The patterns of CNA events mainly include aggregation and dispersed and complete spatial randomness (CSR). To compute the pattern to which the CNA events of genes belong, we random samples N spots in the space 1000 times so that the expected value of K-function from multi-distance can be calculated. Then, two null hypotheses are established as follows.

$$H_{0}^{\left(1\right)}:K\left(observe\right)\;\geq \;K\left(expected\right)\;H_{0}^{\left(2\right)}:K\left(observe\right)\;\leq \;K\left(expected\right)$$

If the first is rejected, the CNA event of genes is a dispersed pattern. If the second one is rejected, the CNA event of genes is an aggregation pattern. Otherwise, the CNA event of genes is a complete spatial randomness (CSR) pattern, which indicates that CNA events occur randomly in the whole space without spatial correlation.

### 2.3. Proof with Simulation and Actual Data

Two simulated and four real spatial transcriptome data were used in our experiments. For the simulation datasets, we first selected breast cancer data obtained by G&T-seq [16], which contains both DNA and transcriptomic data from the breast cancer line HCC38. We calculated the CNA profiles based on genomic DNA by cyclic binary segmentation (CBS) analysis [17] and obtained the CNA status of each gene on the chromosome segment based on the CNA results. We used the CNA results for each gene as our ground truth. The second simulation dataset is from the bulk data of relevant tumor cells in TCGA. The DNA and transcriptome of the same tissue sample of the same patient were sequenced separately. The method used for obtaining ground truth was the same as the previous experiment. The spatial distribution corresponding to each sample of these datasets is generated by the novoSpaRc [18] method (Supplementary Note S1). The four real spatial transcriptome data include human breast cancer, skin squamous cell carcinoma, head and neck square cell carcinoma and lung cancer based on the mouse genome. The data preprocessing is in Supplementary Note S2.

## 3. Results

### 3.1. Applying CVAM to High-Resolution Simulated Spatial Transcriptomic Data

**Figure 3:**
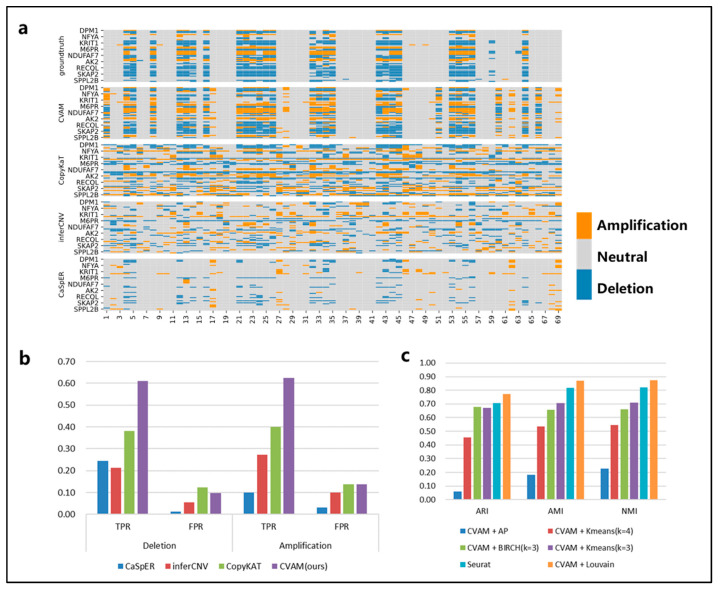
The results of CVAM on the high-resolution simulated spatial transcriptome. (**a**) Copy number alteration profiles inferred by different tools. The horizontal axis is spot id, the vertical axis is gene id, orange represents amplifications, grey represents neutral, and blue represents deletion. (**b**) Comparison of different tools f©CNA inference. (**c**) Comparison of different clustering methods. The specific comparison of different clustering algorithms on ARI, AMI and NMI clustering indicators.

We applied CVAM to the simulated spatial transcriptome data based on the breast cancer line HCC38 and the B lymphoblastoid line HCC38-BL from G&T-seq (Figure 3). Compared with the other three tools, CVAM improves the TPR (61.09% TPR of deletion and 62.51% TPR of amplification) of inferring CNA events (Figure 3b and Table S1). Moreover, the visual presentation of © profiles (Figure 3a) suggests that the inference results of CVAM are closer to the ground truth. In addition, we also tested the impact of different clustering methods on the clustering results (Figure 3c, Supplementary Figure S1). Through the Adjusted Rand Index (ARI), Normalized Mutual Information (NMI) and Adjusted Mutual Information (AMI), whose definition and computation are shown in Supplementary Note S3, we can find that the CVAM with the Louvain algorithm performs better than others. CVAM utilizes VGAE to perform nonlinear dimensionality reduction on gene expression matrices. Compared with Seurat’s PCA linear dimensionality reduction, CVAM has a better generalization ability. In addition, before using the Louvain unsupervised algorithm for clustering, Seurat only used the KNN algorithm to construct the graph. In contrast, CVAM further uses the coordinate information of each spot in the actual space to correct the graph so that the gene expression is similar and the adjacent spots in the space are more closely connected in the graph, thereby improving the clustering performance.

### 3.2. Applying CVAM to Simulated Spatial Transcriptome Data from Bulk RNA-Seq

**Figure 4:**
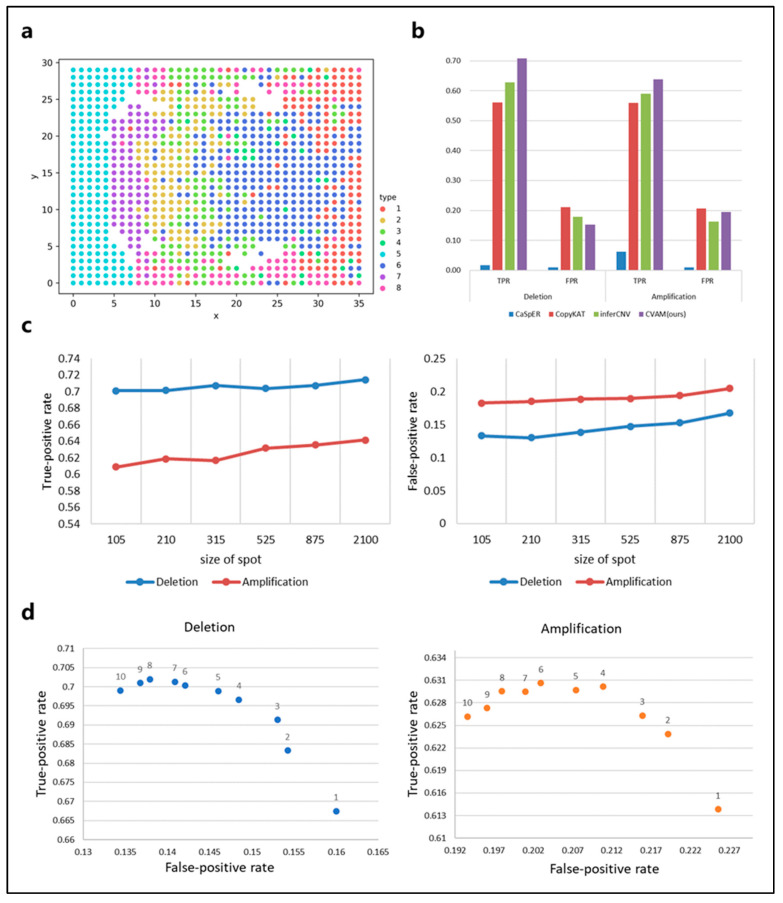
The results of CVAM on simulated spatial transcriptome data from bulk RNA-seq. (**a**) The clustering distribution of spots in space. The simulated spatial transcriptome sample clustered into eight types. (**b**) Comparison of different tools for CNA inference in the data. (**c**) The performance of CNA events identification under CVAM with different sizes of the spot. (**d**) The performance of CNA events identification under CVAM with different window scales.

We also applied CVAM to the simulated spatial transcriptome data from bulk RNA-seq (Figure 4). Figure 4a shows the spatial distribution profile of the simulation data and the clustering results. In this dataset, CVAM achieves 70.75% TPR with 15.24% FPR in gene deletions and 63.79% TPR with 19.46% FPR in gene amplifications (Figure 4b and Table S2). In order to explore the influence of spot size on the results. We randomly sampled datasets with different spot sizes and conducted experiments separately. The results are shown in Figure 4c. We can see that as the spot size continues to increase, TPR and FPR both show an upward trend. However, the trend of changes in TPR and FPR of CNA is not significant. As the spot size increases, HMM’s transfer probability and emission probability become more accurate. However, due to the high degree of convergence of model parameter training, it has little impact on the final results. In addition, the increase in spot size also leads to an increase in outliers. However, the multi-scale median filtering smooths the abnormal mutation values of gene expression and improves the model robustness so that the results are not worsened. These experiments have shown that spot size has relatively little influence on the results, reflecting the universal application of our method under different spot sizes. In addition, we also compared the performance under the different scales of multi-level median filters (Figure 4d). At smaller scales, the model has limited ability to identify CNA events, while the performance is better at a large scale.

### 3.3. Applying CVAM to the Spatial Transcriptome Data of Breast Cancer

**Figure 5:**
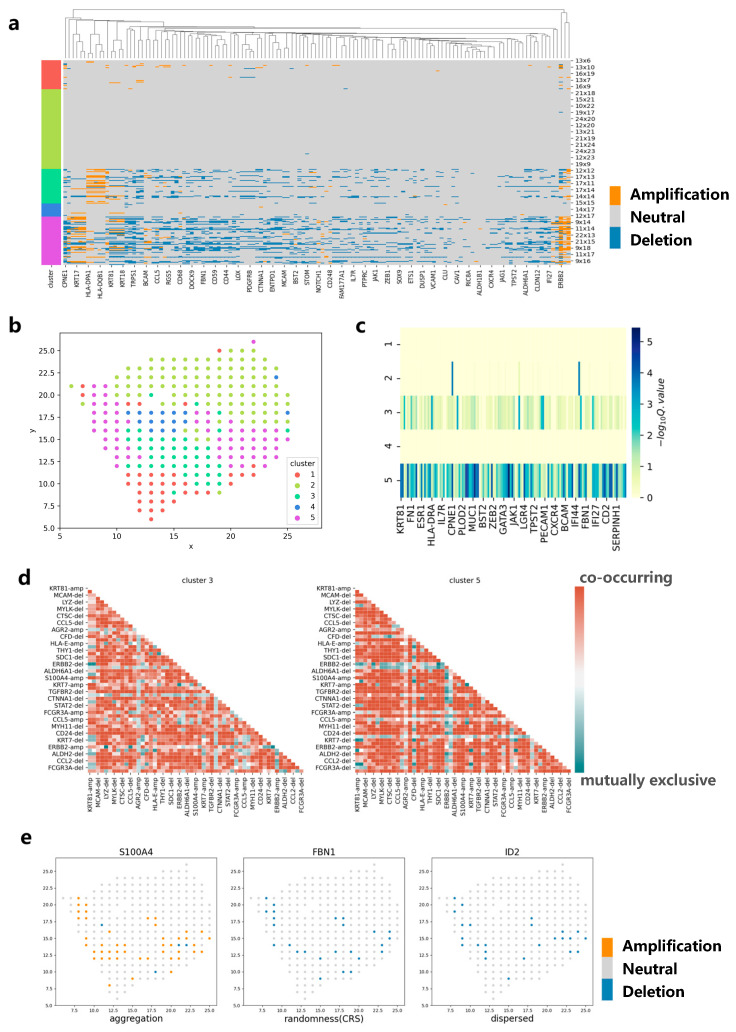
Applying CVAM to the spatial transcriptome of breast cancer. (**a**) CNA profiles inferred by CVAM. The color blocks on the left show the types of spots. The labels on the right show the spatial information. (**b**) The cluster results in space. (**c**) Differential gene profiles by analyzing the CNA events in the different clusters. The darker the color, the more significant the CNA difference of genes in the tumor cluster compared with normal spots. (**d**) The mutually exclusive and co-occurring CNA events profile of cluster 3 and cluster 5. The darker the color, the more significant the mutually exclusive or co-occurring CNA events. Red means co-occurring while green means mutually exclusive. “-amp” means the amplification event while “-del” means deletion event. (**e**) The CNA distribution of genes with different spatial patterns. Left is the S100A4 with aggregation pattern (d = 4, *p* < 1 × 10^−38^) while right is ID2 with dispersed pattern (d = 4, *p* < 1 × 10^−5^) and center is FBN1 with complete spatial randomness (CRS) pattern.

We apply CVAM to analyze the spatial transcriptome data of breast cancer (Figure 5). The spots in this dataset are mainly divided into five clusters (Figure 5b). Analyzing the marker genes [19] in each cluster, we find that cluster 3 and cluster 5 are tumor cell clusters. The CNA profiles show that high-frequency CNA events mainly occur in clusters 3 and 5 (Figure 5a). In the two clusters, S100A4, ERBB2, KRT19 and other genes have occurred copy number amplification, while ACTA2, FBN1, ID2 and other genes have occurred copy number deletion. These results have been recorded in detail in the cBioPortal [20] database related to breast cancer. In addition, CNA distributions also show that the spots belonging to cluster 3 or cluster 5 are more different from the normal spots (Figure 5c). By comparing the co-occurrence and mutually exclusive analyses of cluster 3 and cluster 5, we can also find the potential relevance of CNA between genes in different clusters (Figure 5d). For example, the amplification of S100A4 and the amplification of KRT81 exhibit a co-occurrence relationship in both clusters, indicating that they have a common impact on tumor development and that there is a potential concomitant occurrence between the two genes. In the spatial pattern, it can be found that the genes such as S100A4 show an aggregation pattern (d = 4, *p* < 1 × 10^−38^) while ID2 shows a dispersed pattern (d = 4, *p* < 1 × 10^−5^) and FBN1 shows a complete spatial randomness pattern (Figure 5e). This reflects the differences in CNA spatial patterns among different genes, which can help implement more effective treatment plans for the spatial characteristics of different genes in the future.

### 3.4. Applying CVAM to the Spatial Transcriptome Data of Skin Squamous Cell Carcinoma

**Figure 6:**
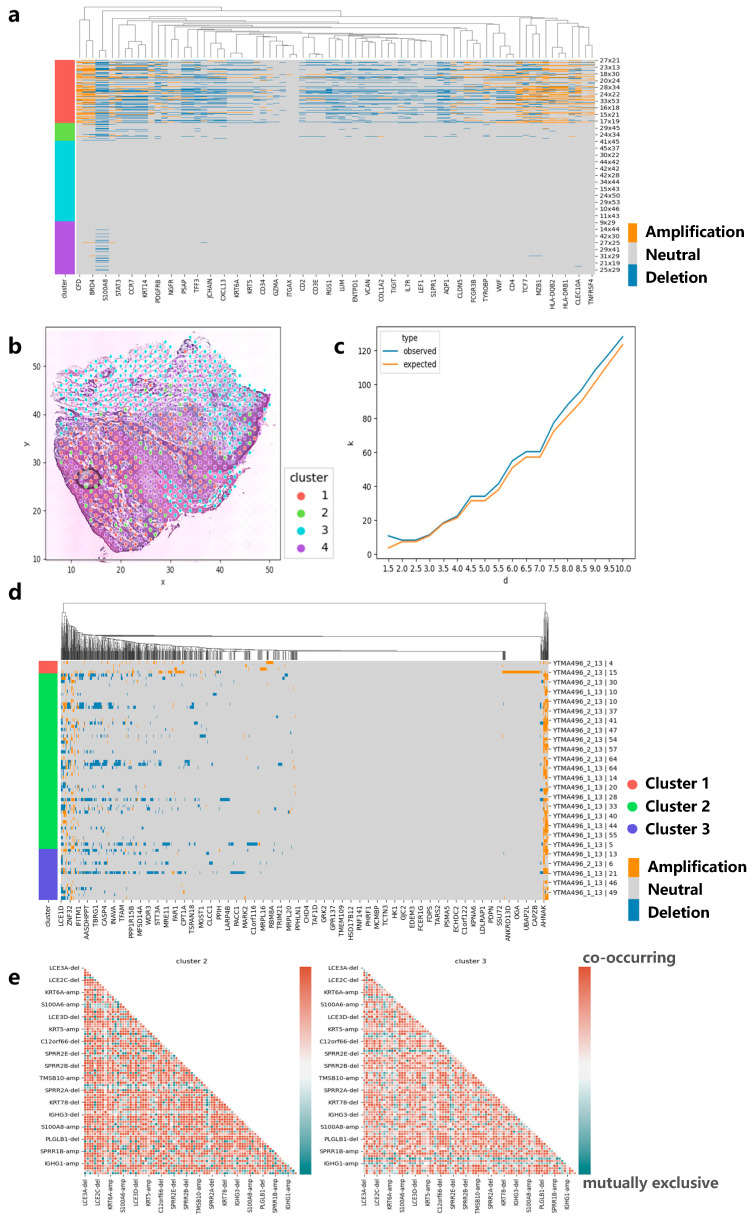
Applying CVAM to the spatial transcriptome of squamous cell carcinoma. (**a**) CNA profiles of skin squamous cell carcinoma inferred by CVAM. (**b**) The cluster results of skin squamous cell carcinoma in space. (**c**) The K-function values of gene EIF1AD with the increase in the distance **d**. Blue is from observed spots, while orange is from the random sampling expected results under the complete spatial randomness pattern. (**d**) CNA profiles of head and neck square cell carcinoma inferred by CVAM. (**e**) The mutually exclusive and co-occurring CNA events heatmap of cluster 2 and cluster 3.

We also apply CVAM to a spatial transcriptomic dataset of human skin squamous cell carcinoma (Figure 6a). Similarly, we found that genes such as CD7, CD1A, and HLA-DPB1 experienced significant copy number amplification, while genes such as CD3E experienced significant copy number deletion. These results also can be found in the cBioPortal. Moreover, the co-occurrence and mutual exclusion of genes CNA states also can be found. The pairs such as HLA-DRB1-amp/PDGFRB-amp showed co-occurrence in cluster 1 (*p* < 1 × 10^−5^), while pairs such as BRD4-del/KLF2-amp show a mutually exclusive (*p* < 1 × 10^−5^). Finally, we also found genes with CNA event spatial patterns. Genes such as EIF1AD present CRS patterns on a small scale, indicating that CNA events for such genes exhibit randomness in local space. As the region expands, the observed K-value is above the expected K-value, which reflects the CNA events showing that aggregation patterns occur more intensively (Figure 6c).

### 3.5. Applying CVAM to the Spatial Transcriptome Data of Head and Neck Square Cell Carcinoma

In order to test the performance of our method on different sequencing platforms, we also applied CVAM to the spatial transcriptome data of head and neck square cell carcinoma based on digital spatial profiling (DSP) of Nanostring (Figure 6d). We observed a large number of CNA amplifications in genes such as S100A11, S100A2, KRT16, and KRT17 in cluster 2 and cluster 3, while genes such as KRT78, IGHG4, and IGHG2 exhibited a large number of CNA deletion events. They are consistent with the results in the cBioPortal. Through co-occurrence and mutual exclusion, we found that the two clusters have some differences in CNAs; for example, S100A12 and S100A16 amplifications significantly co-occurred in cluster 3 (*p* < 1 × 10^−3^, FDR < 0.05), while no correlation was found in cluster 2. However, in cluster 2, the amplifications of S100A10 and AHNAK genes were significantly co-occurrence (*p* < 1 × 10^−3^, FDR < 1 × 10^−2^). Both genes participated in the regulation of cell membrane cytoarchitecture [21], which also reflected the CNA differences between clusters (Figure 6e).

### 3.6. Applying CVAM to the Spatial Transcriptome Data of Lung Cancer

**Figure 7:**
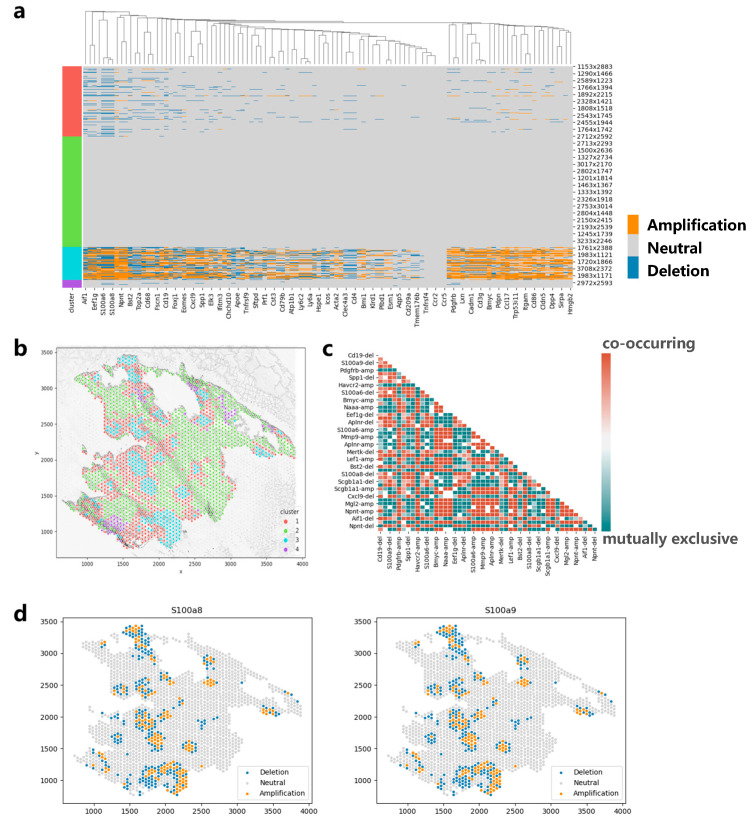
Applying CVAM to the spatial transcriptome of lung cancer. (**a**) CNA profiles inferred by CVAM. (**b**) The cluster results in space. (**c**) The heatmap of mutually exclusive and co-occurring CNA events. (**d**) The CNA distribution of genes (S100a8 and S100a9) with the spatial pattern.

We also apply CVAM to a spatial transcriptomic dataset of lung cancer based on a mouse model (Figure 7). The CNA results and clustering results are shown in Figure 7a and Figure 7b, respectively. It can be found that genes such as S100a6, S100a8, and S100a9 showed significant amplification in cluster 3. These genes were reported to be highly expressed in lung cancer in the previous studies [22,23], which may be due to copy number amplification leading to tumor growth and metastasis in related cancer cells. In addition, through the co-occurrence and mutual exclusion analysis (Figure 7c), we also found that the amplification of S100a8 and S100a9 exhibits a co-occurrence relationship (*p* < 1 × 10^−79^). The consistent impact of these two genes on lung cancer metastasis has also been reported in a previous study [24]. In terms of spatial pattern, we found that the CNAs of S100a8 and S100a9 both exhibit aggregation patterns locally (d = 50, *p* < 1 × 10^−5^) and mainly aggregate in cluster 3 (Figure 7d). From global space, it is more inclined towards a discrete distribution pattern (d = 150, *p* < 1 × 10^−20^). In particular, from the figure below, we can also find some copy number deletion spots in the tissue, because these spots are mainly located in the cytoplasm or ST sequencing background region, and the measured gene expression values are much smaller than that of normal cells.

## 4. Discussion

The CNA profile plays an important role in the analysis of polyclonal characteristics of tumor cells. We propose a CNA profile inference tool, CVAM, for analyzing genetic variants from spatial transcriptome data. We first cluster the samples based on the feature similarity and spatial characteristics through VGAE. Compared with the traditional PCA linear dimension reduction, the generalization ability of CVAM is higher and the samples can be divided more correctly. In addition, compared with the simple VAE [25,26], VGAE integrates spatial information with the spot’s gene expression information together so that the heterogeneous information of samples can be analyzed, which is conducive to the unsupervised clustering between adjacent spots.

In CNA inference, multi-level filters can remove the interference of outliers and the window size of filters. Compared with CaSpER, CVAM performs multi-scale integration before HMM, which reduces the time cost of model training. Although inferCNV using Bayesian and HMM performs relatively well, it only performs better on consistent CNA inference under a large number of samples, and the performance on higher-resolution data remains to be improved. Compared with these existing tools, our experimental results demonstrate that CVAM performs better in terms of accuracy and TPR for CNA events. In addition, although our tool is developed for spatial transcriptome data, after reconstructing the spatial distribution of samples through an optimal transmission algorithm, this method is also applicable to single cells or bulk transcriptome sequencing data. Moreover, through co-occurrence and mutual exclusion analysis, more and more potential connections between genes will be revealed, which will help us to further explore the molecular mechanism of tumor development and variation. Last but not least, gene spatial pattern analysis is one of the most important analysis methods in bioinformatics after the emergence of the spatial transcriptome. With the continuous development of spatial transcriptome technology, there will be more and more exploration of the spatial characteristics of tumor development in the future, which is important for the design of drugs and treatment plans.

There are also some limitations and shortcomings in the CVAM. The results are gene-level CNA profiles. So, the resolution of CNA can be further improved. Moreover, the number of states of CNA can increase, making the measurement of CNA events more detailed. In future works, we will further explore the detailed and effective division of chromosome sequence so that the resolution and accuracy of this method can be improved.

With the development of slide-DNA-seq [27] that can capture spatially resolved DNA sequences, it is believed that more research will be devoted to the joint analysis of spatial transcriptome and genome data in the future. This will help to further understand the heterogeneity of tumors and provide some auxiliary decision-making for the precise treatment of related tumor diseases.

## Data Availability

All the data in this paper are publicly available. The G&T-seq data are derived from [16]. Normal breast single-cell data can be accessed from the Gene Expression Omnibus (GEO) databases with accession numbers GSE113197. Pan-cancer data are derived from [28] and the normal breast bulk RNA-seq data can be accessed from TCGA-BRCA. The spatial transcriptome data of breast cancer are from [29], the spatial transcriptome data of skin squamous cell carcinoma are from [30], the spatial transcriptome data of head and neck squamous cell carcinoma are from [31] and the spatial transcriptome data of lung cancer are from [32]. Code availability: CVAM is written in Python language (v3.8.5). The code has been open source and analysis results are listed publicly at GitHub: https://github.com/JMnotDGR/CVAM (accessed on 25 April 2023).

## Supplementary Materials

The following supporting information can be downloaded at: https://www.mdpi.com/article/10.3390/biom13050767/s1, Note S1: Generation of simulation spatial transcriptome data; Note S2: Gene expression data preprocessing; Note S3: Evaluation; Figure S1: The clustering distribution of spots under different clustering algorithms; Table S1: The evaluation result of CVAM on high-resolution simulated spatial transcriptome; Table S2: The evaluation result of CVAM on simulate spatial transcriptome data from bulk RNA-seq.
