# Supplementary material for: CVAM: CNA Profile Inference of the Spatial Transcriptome Based on the VGAE and HMM
Source: Biomolecules. 2023 Apr 28;13(5):767. doi: 10.3390/biom13050767 (PMC10216626; doi:10.3390/biom13050767)
Supplement: Supplementary file 1 [file biomolecules-13-00767-s001.zip › biomolecules-2320692-supplementary.pdf]

Supplementary of:

CVAM: CNA Profile Inference of the Spatial Transcriptome Based on the VGAE and HMM

Note S1

Generation of simulation spatial transcriptome data

The corresponding spatial distribution of each cell is simulated by novoSpaRc. First, give a 2D or 3D target physical space (e.g., 20×20 rectangle in 2D space), and then the graph of physical space is generated based on the distance between sites. Each site corresponds to a node. If the distance less than threshold, we consider that there are an edge connecting the two nodes. Subsequently, based on the gene expression characteristics of single cells, construct a k-nearest neighbors graph, and each node represents a cell. Because cells have similar relative distances from other cells in gene expression and physical space, based on these two graphs, we can find a probabilistic mapping that assigns each cell a distribution over locations on the physical space through the optimal transmission algorithm. With the probability of each cell in each position in a given space, we can find a joint optimal allocation under the constraint that each cell can only be assigned to an independent location. Since the complexity of the algorithm for solving the maximum joint probability is O(nm), in order to reduce the time complexity of the algorithm, we use greedy algorithm to assign each sample cell to the position with the greatest probability one by one. In other words, for a cell, if and only if there is no allocated cell at the position, the cell is allocated to the spatial position. Otherwise, except for the position, calculate the next spatial position with the maximum probability of the cell, and so on. Finally, the distribution result of each cell in a given space can be obtained. Each spot corresponds to a single cell. We can regard it as spatial transcriptome data with higher resolution compared to the real spatial transcriptome data which contains 5~20 single cells per spot.

The G&T sequencing technology can simultaneously sequence genomic DNA and full-length mRNA from single cells. Therefore, based on the genomic DNA, the ground truth of copy

number profiles can be computed through $2^{\log R} \times \Psi$, where the average ploidy of the cell, Ψ, was estimated based on the logR value of a large reference region with known DNA copy number. As usual, the values were further thresholded by a noise cutoff of 0.3: smaller than -0.3 are categorized as "deletion", larger than 0.3 are categorized as "amplification", others are categorized as "neutral".

Note S2

Gene expression data preprocessing

The input of CVAM is the gene expression matrix of spatial transcriptome X, $X \in R^{m\times n}$, m is the number of genes in each spot, and n is the number of spots. For the gene expression matrix X, the following three preprocessing steps are required. The first step is to convert the original count expression matrix into the TPM value, so as to eliminate the influence of sequencing depth on the subsequent analysis process. The second step is to filter the low expression genes. For the whole corrected gene expression matrix, set the threshold α (default set as 5%), and the genes expressed in less than α of all spots will be filtered. The third step is to eliminate the batch effect between samples from different batches in the dataset. If the sequencing data are from the same batch or there is no batch effect, this step can be ignored. We use X' to represent the preprocessed gene expression matrix obtained after three steps.

In the clustering stage, in order to minimize the influence of genes with not significant difference to clustering, it is also necessary to set the variance threshold σ (default set as 1). Filter out the low variant genes in the gene expression data, so that the subsequent process can mainly use the differentiation characteristics between spots to learn the low-dimension representation of spots, and the filtered gene expression matrix is represented by X''. In the CNV profile inference stage, to retain the expression information of the original gene in the chromosome sequence as much as possible and improve the effectiveness of the median filters, no redundant filtering processing is done and only compute the library-size log-normalized gene expression matrix X^l. Subsequently subtract the gene expression data of normal spots from X^l, namely $\hat{X} = X^l - X^l_{normal}$, which is used to infer the variation states of genome copy number.

Note S3

Evaluation

We evaluate the unsupervised clustering performance through Adjusted Rand Index, Normalized Mutual Information and Adjusted Mutual Information.

The Adjusted Rand Index (ARI) is expressed as:

$$ARI = \frac{RI - E(RI)}{\max(RI) - E(RI)}$$

Here, $RI = \frac{a+b}{C_n^2}$, a is the pair number in the same cluster of the label and the prediction result, while b is the pair number in the same cluster of the label but not in the same cluster of the prediction result. $E(RI)$ is the expected value of RI.

The Normalized Mutual Information (NMI) and Adjusted Mutual Information (AMI) are expressed as:

$$NMI(U,V) = 2\frac{MI(U,V)}{H(U) + H(V)}$$

$$AMI(U,V) = \frac{MI(U,V) - E[MI(U,V)]}{\frac{1}{2}(H(U) + H(V)) - E[MI(U,V)]}$$

Here, U is the real label set, V is the predicted label set. $MI(U,V)$ is the mutual information between the two sets. $H(U)$ and $H(V)$ are the information entropy of the two sets, respectively, and $E[MI(U,V)]$ is the expectation of mutual information.

For the CNV profile, we mainly evaluate them from three aspects: accuracy, TPR and FPR. The accuracy is the percentage of correct genes predicted by the copy number state in the total genes. TPR is the percentage of genes with a copy number state correctly identified in all genes with CNV events, while FPR is the percentage of genes with a copy number state incorrectly inferred in all genes without CNV events. According to the above definition, the calculation method can be expressed as:

$$Accuracy = \frac{1}{n \times m} \sum_{i=1}^{n} \sum_{j=1}^{m} S_{ij}\ ,\quad S_{ij} \begin{cases} 0, & prediction\ wrong\ of\ gene\ j\ in\ spot\ i \\ 1, & prediction\ right\ of\ gene\ j\ in\ spot\ i \end{cases}$$

$$TPR = \frac{TP}{TP + FN}$$

$$FPR = \frac{FP}{FP + TN}$$

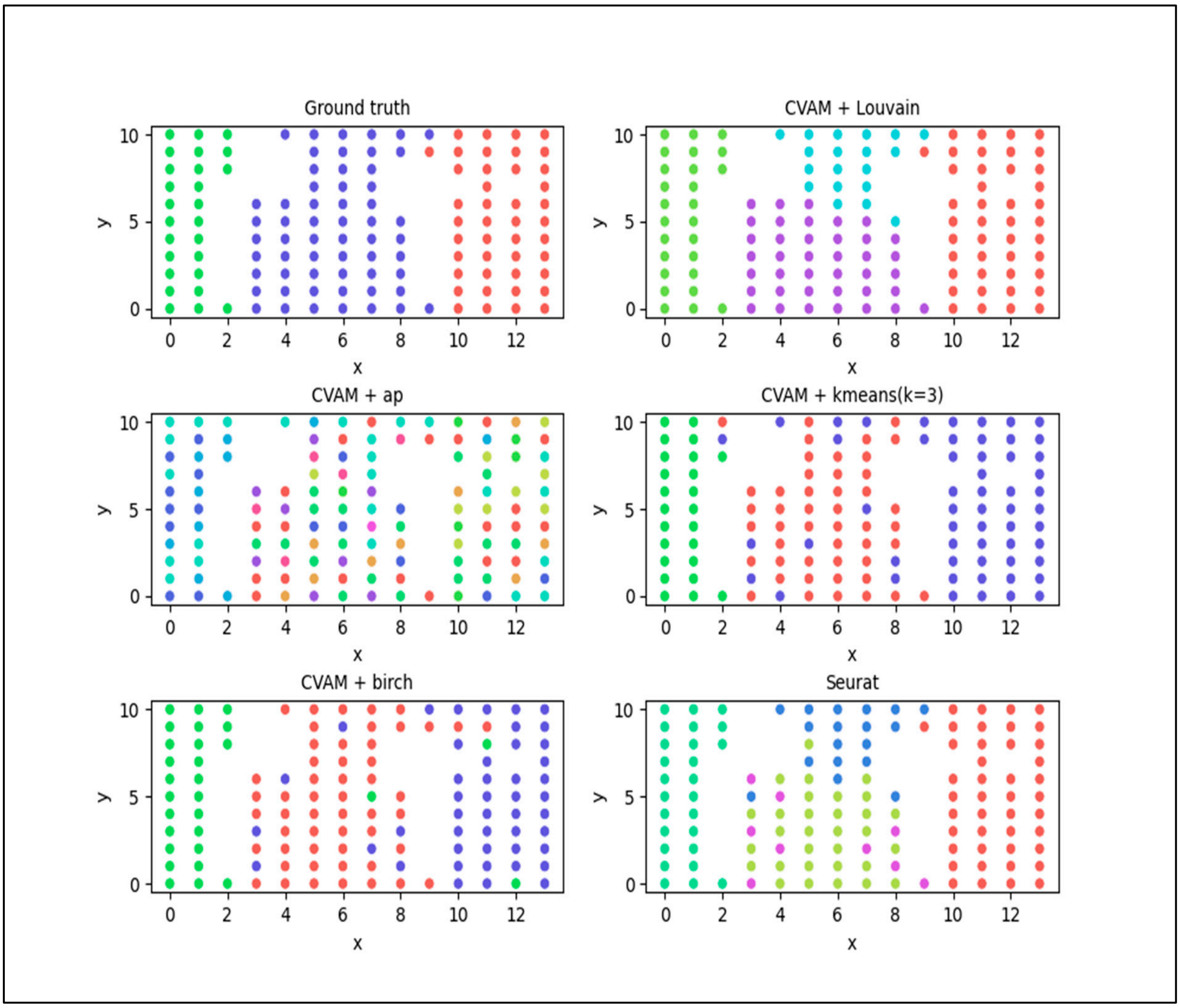

Figure S1. The clustering distribution of spots under different clustering algorithms. The results of unsupervised clustering algorithms: Louvain, Affinity propagation (AP), KMeans, Birch and the result of Seurat. The X and Y axes are the corresponding positions of spots in two-dimensional space.

Table S1

The evaluation result of CVAM on high-resolution simulated spatial transcriptome

GT	Deletion		Amplification		Accuracy
	TPR	FPR	TPR	FPR	
inferCNV	21.35%	5.60%	27.28%	9.99%	68.18%
CopyKAT	38.13%	12.49%	40.08%	13.92%	62.97%
CaSpER	24.51%	1.30%	10.13%	3.20%	76.31%
CVAM(ours)	61.09%	9.85%	62.51%	13.76%	73.14%

Table S2

The evaluation result of CVAM on simulate spatial transcriptome data from bulk RNA-seq

BRCA	Deletion		Amplification		Accuracy
	TPR	FPR	TPR	FPR	
inferCNV	62.80%	17.89%	58.94%	16.22%	62.07%
CopyKAT	56.11%	21.10%	56.00%	20.70%	54.87%
CaSpER	1.73%	1.00%	6.25%	1.00%	80.19%
CVAM(ours)	70.75%	15.24%	63.79%	19.46%	62.43%
